# Volume Twelve

**Published:** 1895-03

**Authors:** 


### EDITORIAL.

The office of THE JOURNAL is in room 330 Equitable Building.

The editors are not responsible for views expressed by contributors.

Articles for publication should reach this office not later than the 15th instant.

Address all communications, and make all remittances payable to THE ATLANTA MEDICAL AND SURGICAL JOURNAL, Atlanta, Ga.

Reprints of articles will be furnished, when desired, at a nominal cost. Requests for the same should always be made *on the manuscript*.

### VOLUME TWELVE.

This issue is the beginning of our twelfth volume, new series. Up to March, 1884, the career of this JOURNAL had been more or less uncertain, alternating between periods of promising vitality and states of suspended animation. At the above date it was thoroughly and firmly re-established, and placed upon such a sure foundation that it has since enjoyed the longest period of continuous publication in its history. Since that date THE JOURNAL has aimed at constant and progressive improvement, and the steady growth in our business, both subscription and advertising, has been a gratifying encouragement.

THE JOURNAL has never been in better condition than it is today; and, in beginning our twelfth year of publication, we assure our patrons that we will seek to improve it still further. To this end we again invite the active coöperation of our readers and friends everywhere.

